# Research on the aesthetic sensitivity evaluation of tourism mascots based on semantic differential method

**DOI:** 10.1371/journal.pone.0318715

**Published:** 2025-02-14

**Authors:** Jing Wang, Wenting Yao, Sheng Su, Jing Zhang, Li Wang

**Affiliations:** School of Design, Xi’an Technological University, Xi’an, China; Jiangnan University, CHINA

## Abstract

In order to better meet the emotional needs of consumers and enhance the design of mascots that integrate multicultural elements, this study uses sensory engineering theory to explore the correlation between emotional needs and aesthetic forms. Initially, tourism mascot forms were collected through market surveys, and then these forms were synthesized into test samples. At the same time, expert interviews were conducted to determine the adjective pairs that can accurately capture consumers’ emotional needs. Subsequently, tourists who came to Xi’an were invited to participate in a satisfaction survey. Using the semantic differential methods, representative adjective pairs were selected from five dimensions: market positioning, market trends, color decoration, stylistic features, and psychological perception as emotional evaluation criteria. Each tourism mascot was evaluated and rated according to these criteria. SPSS software was then used to conduct a one-way ANOVA on the evaluation scores to explore the relationship between the aesthetic evaluation of various mascots and consumers’ emotional needs. The experimental results show that the IP image of cultural and creative brands designed with Tang cultural elements is more contagious and avant-garde than the IP image of cultural and creative brands that incorporate Qin cultural elements. These designs can resonate more with young consumers. This study successfully establishes a mapping model that combines consumers’ aesthetic preferences with their emotional needs for tourism mascot design. This study provides valuable guidance for the design of Xi’an tourism mascots and effectively meets consumers’ emotional expectations.

## 1. Introduction

In recent years, the design of cultural and creative product has gradually shifted to being consumer-centric, incorporating story content [1], and using media such as brands, mascots, and the Internet to get closer to young consumers [2], giving rise to. a number of excellent brands and mascots. Although the current market is still dominated by big IPs and big brands, with the support of the Internet and the increased IP protection, innovation and creativity will lead future market trends [3]. In this context, the combination of regional culture and cultural and creative products tends to be diversified and three-dimensional, but it still faces problems such as talent shortage and design homogeneity. One of the core challenges is how to effectively combine regional culture with innovative products and realize industrialization [4]. This study takes the Xi’an regional cultural tourism mascot as an example and analyzes the advantages and disadvantages of its cultural and creative brand tourism mascot design from the perspective of aesthetics evaluation [5]. It aims to solve the problems in consumer aesthetic evaluation, promote the dissemination of Xi’an’s regional culture, and provide theoretical support for the innovative development of mascot design in the cultural tourism industry.

The term “regional culture” originally refers to a cultural characteristic formed in a relatively certain geographical area, reflecting the distinctive characteristics of the region [6]. Yu Xiaoqun defines regional culture as a branch of geography and humanities, which studies the spatial aggregation of human civilizations in different locations and has obvious regional characteristic. In some aspects, regional culture can be regarded as a synonym for cultural geography, which studies cultural phenomena such as cultural thoughts, natural and cultural landscapes in a specific region [7,8]. In addition, regional culture also includes historical relics, cultural forms, customs, production and living styles, etc. in a specific geographic area. Regional culture can be divided into material culture, behavioral culture, and psychological culture. Material culture is the tangible part of regional material culture that exist in an objective, physical state. It is a unique regional symbol that records the formation and evolution of culture [9], such as landmark buildings, cultural relics, handicrafts, and traditional costumes. Behavioral culture is the crystallization of wisdom formed by regional residents in their labor practice, including elements such as language, writing, and traditional crafts [10]. Psychological culture refers to the unique spiritual outlook, psychological characteristics, and conceptual consciousness formed by the residents of a region in social practices and conscious activities.

Cultural and creative products, as defined by the United Nations Educational, Scientific and Cultural Organization (UNESCO), are consumer goods that embody creative ideas, symbols, and lifestyles [11]. They are the carriers of the core values of cultural and creative brands, establish a unified brand image in the minds of consumers, and are the key factors affecting purchase intentions [12]. Brand mascots not only serve as a distinctive identifier for cultural and creative brands but can also effectively spread brand values through multiple channels and enhance brand awareness [13]. The scalability of mascots allows for a more flexible and impactful transmission of the brand’s core values, while their unique personalities contribute significantly to shaping and amplifying brand influence, thereby establishing them as super symbols in brand identity [14].

Tourism mascots are an integral part of the cultural and creative industries and play a vital role in shaping a unique brand image and enhancing brand reputation. In addition to these functions, mascots also help to increase the added value of products, enrich product lines, and enhance market competitiveness [15]. In the context of the cultural tourism industry, strategically focusing on the development and promotion of mascot images is essential to ensure a larger market share and maximize economic benefits.

Xi’an, historically known as “Chang’an” and “Jingzhao,” is one of the four ancient capitals of civilization in the world. It is the capital with the longest history and the most far-reaching influence in Chinese history. If the regional culture is classified according to material culture, behavioral culture and psychological culture, then Xi’an’s material culture includes the ancient city wall, the Big Wild Goose Pagoda, the Bell Tower, the Tang Paradise, the Qin Terra Cotta Warriors, and the Tang Terracotta Warriors; behavioral culture includes shadow puppetry, the Chang’an painting school, Shaanxi puppetry, Huxian peasant painting, and Xi’an drum music; psychological culture includes cultural customs of various dynasties, including the Zhou, Qin, Han, and Tang.

Although numerous studies have emphasized the significance of tourism mascots in the cultural tourism industry, there are a relative few studies on the aesthetic evaluation of tourism mascot design. Existing studies often rely on qualitative analyses or single-indicator approaches, which are unable to obtain comprehensive sensory evaluation results[15]. In the design and evaluation of mascots, a method that combines subjectivity and objectivity must be adopted, taking into account consumers’ emotional reactions and resonance while ensuring the scientificity and objectivity of the evaluation process. To accurately capture consumers’ emotional reactions and resonance, designers must conduct in-depth market research and consumer analysis, understand market positioning, trends, consumer preferences and values, and then design mascots that effectively cater to consumers’ emotional needs. This study will explore the optimization of urban tourism mascot design under the regional cultural background of Xi’an, and use the semantic differential method combined with aesthetic evaluation for in-depth analysis [3].

The Semantic Differential is a psychological measurement tool that converts qualitative perceptions into quantitative data by evaluating adjectives pairs. Consumers are asked to rate these adjective pairs based on their subjective feelings [16]. The data collected from these evaluations provide designers with insights into consumers’ emotional tendencies and design preferences, thereby optimizing product design [17]. This method improves the reliability and comprehensiveness of measurement items to a certain extent [18]. We believe that the semantic differential scale can reveal multidimensional aspects of mind perception [19]. This scale does not involve questions about mental ability, but can assess subject’s non-verbal impressions of various objects, events, and concepts based on the degree of matching of multiple adjectives and entities [20]. In this method, an adjective is paired with its antonym, and the two adjectives are assigned numbers on the scale. For example, “cold” and “warm” are paired and assigned 1 and 7 respectively. Subjects are asked to rate the entity on the scale [21]. Questionnaires on paired adjectives showed that mind perception differed in two dimensions of mind, namely mind holding ability and mind reading ability. It can be regarded as an effective method to intuitively assess the multidimensional aspects of mind perception [20]. This method enhances the reliability and comprehensiveness of the measurement items to a certain extent. The Semantic Differential method is widely applied in various disciplines, such as furniture design [22], landscape design [18], architectural design [23], and product design, and can effectively evaluate consumers’ emotional responses to products or designs. In the context of tourism mascot design, the Semantic Differential method can provide a deep understanding of the key factors that influence consumer choices. By analyzing the data obtained, designers can identify shortcomings of existing mascot designs and use this information for subsequent improvements. This approach provides a reliable basis for improving the design elements of Xi’an regional tourism mascots so that they resonate with consumer preferences and emotional needs.

Aesthetic sensitivity is the degree to which an individual responds to aesthetic stimuli based on consistency and appropriateness with external standards [24]. Visual aesthetic sensitivity, as a universal and objective key element in aesthetic appreciation [25,26], reveals individual differences in people’s aesthetic abilities [27–29]. This difference is mainly reflected in the accuracy of individuals’ identification of aesthetic quality [30] and differences in their judgment of the quality of artworks [31]. In the visual aesthetic sensitivity test, individuals who are able to identify, analyze, and evaluate various aesthetic feature defects will show higher aesthetic quality identification ability [28]. Many studies in the field of aesthetic evaluation focus on how specific personality traits intervene and influence the judgement process of specific types of aesthetics [32–35], as well as the relationship between these personality traits and aesthetic-related fields, especially visual arts [34,36,37]. Studies have shown that psychological experiences, such as beauty, surprise and awe can not only enrich an individual’s emotional experience level, but also have a positive association with improved well-being and quality of life [38,39]. Aesthetic sensitivity is the ability to identify aesthetic qualities in various situation and is one of the core concepts in aesthetic studies [37]. Specifically, people with high visual aesthetic sensitivity tend to pay more attention to the high-level features of aesthetic objects and their overall structure, and give higher-than-average importance and evaluation to aesthetic stimuli that are more aesthetically pleasing [28,40]. In contrast, people with low visual aesthetic sensitivity may pay more attention to directly perceptible, discrete elements and objects in aesthetic stimuli [28]. In the field of design, aesthetic sensitivity assessment is an important method for evaluating the aesthetic components of product design, emphasizing visual elements such as appearance, color, and material, as well as the psychological reactions these elements cause in consumers. This evaluation can be conducted through various methodologies, including questionnaires, interviews, and eye-tracking studies. In this study, we will combine the Semantic Differential method to further investigate the influencing factors and quantitative principles of tourism mascot aesthetics. This approach addresses the limitations of quantitative research in this field by measuring consumer evaluations of the aesthetic qualities of Xi’an’s regional cultural tourism mascots. By designing a series of adjective pairs specifically related to aesthetics, this research aims to provide new insights into the aesthetic sensitivity of tourism mascots.

This study takes tourism mascots as the primary research object and uses the Semantic Differential method to construct a comprehensive and accurate sesthetic quality evaluation model. The main contributions of this study are as follows: (1) Establishing a Comprehensive Aesthetic Evaluation Model: at present, quantitative research on tourism mascots often lacks a comprehensive and complete aesthetic quality evaluation method. To address this shortcoming, the study aims to develop a tourism mascot design aesthetics evaluation model based on semantic analysis. This model will provide a multi-dimensional evaluation framework capable of more comprehensively capturing consumers’ different views and attitudes towards mascots. (2) Identification of Visual Aesthetic Challenges and Cultural Influences: The study will identify common problems related to the visual aesthetic quality of tourism mascots and explore the factors that influence the evaluation of this quality. In addition, by investigating the visual evaluation of tourism mascots, the research will reveal regional cultural differences in the evaluation of mascot design aesthetics. This understanding will be conducive to protecting and promoting regional cultural characteristics by targeted improving the visual aesthetic quality of tourism mascots in a targeted manner [41]. (3) Consumer-Centric Evaluation and Design Optimization: Direct consumer evaluations will intuitively reflect consumers’ preferences and dissatisfaction with tourism mascots. This feedback will provide developers with important insights into the real needs of tourists, thereby improving mascot design in a targeted manner. Such improvements are crucial for increasing tourist satisfaction and experience, thereby contributing significantly to the vitality of the local tourism industry and the broader dissemination and influence of local culture.

## 2. Research methodology framework

### 2.1. Theoretical framework

**Fig 1:**
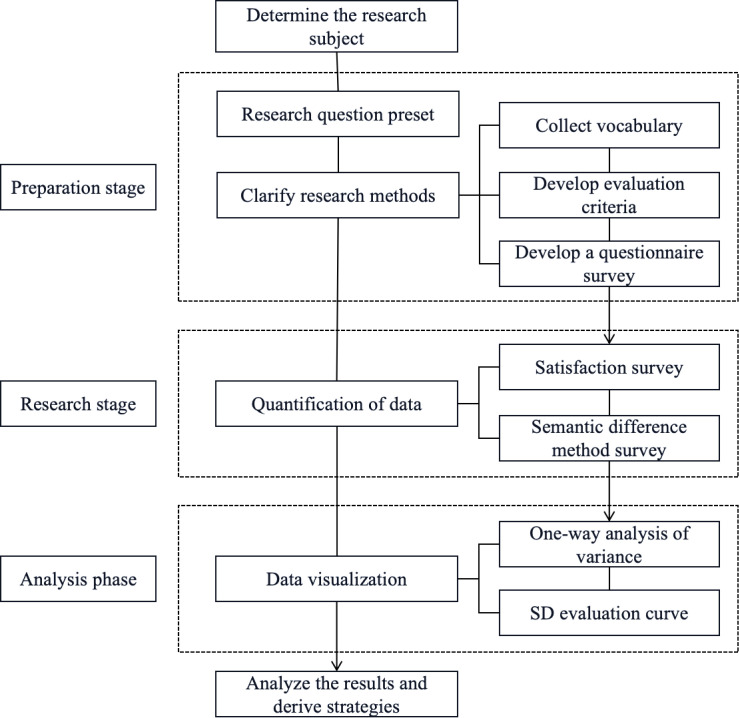
Research method framework.

This study will establish an aesthetics evaluation model based on the Semantic Differential method, serving as the foundational theory for analyzing the design of Xi’an regional culture tourism mascots. The process of developing the tourism mascot aesthetics evaluation model encompasses the following key steps:

The first step involves defining the research object and pre-setting the research questions, laying the groundwork for the subsequent questionnaire survey by compiling relevant vocabulary and developing the evaluation scale. In the second step, a satisfaction survey and a questionnaire using the Semantic Differential method will be conducted to gather data on consumers’ evaluations of the aesthetics of Xi’an’s regional cultural tourism mascot designs. The third step focuses on data visualization, where the evaluation results for each mascot will be generated through one-way ANOVA and Semantic Differential (SD) evaluation curves. The fourth step entails the analysis and interpretation of the collected data, followed by the formulation of targeted design optimization recommendations. This final step also includes validating the aesthetic evaluation model for tourism mascots. The specific process is illustrated in Fig 1 below.

### 2.2. Research subjects

**Fig 2:**
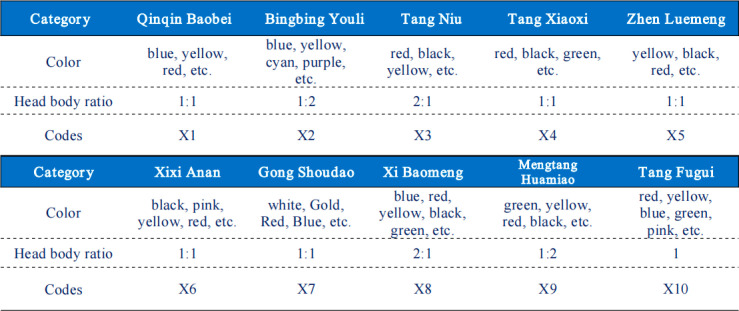
Analysis illustrative of tourism mascots.

To validate the effectiveness of the proposed theoretical framework, we will conduct an empirical study on the design of tourism mascots in the context of Xi’an’s regional culture as a case study. Xi’an, known for its rich and diverse cultural and creative brands, provides an ideal environment for this study. Through actual visits to museums and cultural and creative stores, as well as the collection and research of relevant literature, the top 10 tourism mascots in terms of purchase sales and purchase intention were obtained. This study will focus on the following mascots inspired by Xi’an’s cultural heritage: “Qinqin Baobei,” “Bingbing Youli,” and “Xixi Anan,” which are based on the Qin Terracotta Warriors; “Tang Niu” and “Tang Xiaoxi,” modeled after the figurines of Tang Dynasty ladies; “Tang Xiaodi” and “Tang Xiaofei,” mascots representing the brand Zhen Luemeng, inspired by the verses of the *“Shitai Book of Filial Piety”* by Emperor Xuanzong of the Tang Dynasty, Li Longji; the “City Wall Warriors,” designed after the Datang Imperial Forest Army and featured in the Gong Shoudao series of cultural and creative products developed by Xi’an City Wall; “Tang Minghuang” and “Yang Guifei” from the Xi Baomeng series, which represent mascots of intangible cultural heritage that blend theatrical and modern culture; the “Mengtang Huamiao” painting scroll by artist Guajila, inspired by Tang Dynasty aesthetics; and “Tang Fugui,” based on the horses from the “*Six Steeds of Zhao Mausoleum*”. The specific mascots are depicted in Fig 2.

### 2.3. Collection of evaluation factors

In order to more accurately evaluate the beauty of tourism mascots from an aesthetic perspective, we conducted in-depth research on a number of tourism industry experts from different regions, and finally determined the five dimensions of market positioning, market trends, color decoration, form features, and psychological feelings for evaluation.

In the market positioning dimension, market positioning determines whether tourism mascots can attract the target audiences and effectively convey the brand image and cultural connotations of the tourist destinations. Through accurate market positioning, mascots can better meet the needs of specific groups, especially modern and innovative mascots can increase their competitiveness in the target market.

In the market trend dimension, understanding current market trends is essential for mascot design. This includes analyzing the latest developments in the tourism industry, changes in consumer preferences, and competitor strategies. Keeping up with market trends can help mascots stay up-to-date and relevant and avoid becoming outdated.

In the decorative dimension of color, color is a powerful tool for conveying emotions and information. In mascot design, the choice of color should reflect the characteristics and cultural connotations of the destination while attracting the attention of the target audience. The evaluation of color emphasis needs to consider its visual impact, cultural symbolism, and consistency with the brand image.

In the form features dimension, the mascot’s styling characteristics are the key to its recognizability. A unique and expressive styling can make the mascot stand out from many competitors and become a memorable point in the minds of visitors. When evaluating the styling characteristics, it is necessary to consider its originality, accessibility and fun, as well as the ease of its replication and dissemination in different media.

In the dimension of psychological feeling, mascot design is not only a visual presentation, but also the psychological feeling it brings to tourists. This includes the friendliness and motivation of the mascot. A successful mascot should be able to inspire positive emotions in tourists and enhance their goodwill and interest in the destination.

By determining the aesthetics evaluation dimensions, the selection of evaluation factors is also an important part of this study. Based on the evaluation dimensions, positive and negative adjective phrases that can describe the tourism mascot market, product style, stylistic features, as well as the fit with the destination’s culture and the emotional connection with tourists were collected [15,42–45]. Specifically, the dimensions include: Market positioning (traditional - modern, ordinary - innovate), Market trends (outdated - popular, Rational - emotional), Color decoration (plain - gorgeous, cool - warm, hard - soft), Form features (uninteresting - interesting, complex - simple, vulgar - noble), Psychological feelings (negative - positive, meaningless - meaningful). These dimensions consist of 12 pairs of antonymous adjectives, which serve as the evaluation factors. As presented in Table 1, a 7-point scale ranging from −3 to +3 was selected for this survey to facilitate stability in aesthetic judgment, with scores assigned as follows: −3, −2, −1, 0, 1, 2, and 3. This scale allows for a nuanced assessment of the aesthetic qualities of Xi’an’s regional cultural tourism mascots, providing a balanced measure of both positive and negative perceptions.

**Table 1:** The dimensional evaluation factor adjective pairs.

Dimension	Evaluation factors	Illustrate
Market positioning	Traditional - Modern	Whether the mascot has modern features in its market positioning
	Ordinary - Innovative	Is the mascot innovative in its market positioning
Market trends	Outdated - Popular	Whether the mascot design is in line with contemporary consumer market trends
	Rational - Emotional	Whether the mascot design meets the emotional needs of consumers
Color decoration	Plain - Gorgeous	The mascot has a richer color representation
	Cool - Warm	The mascot has a warmer expression of color
	Hard - Soft	Mascots are decorated in softer colors
Form features	Uninteresting - Interesting	Whether the mascot styling features are interesting
	Complex - Simple	Whether the mascot shape is simple
	Vulgar - Noble	Mascot styling demonstrates whether or not it’s noble
Psychological feelings	Negative - Positive	Does the mascot design give a positive impact on consumers
	Meaningless - Meaningful	Does the mascot design have meaning and value

### 2.4. Survey methodology

Based on the results of the practical survey conducted on “X1” and 10 other cultural and creative brands of tourism mascots, data was collected through online questionnaires. The time is from July 9th to 15th, 2024, with respondents required to spend no less than 150 seconds completing the survey. Before conducting the research, voluntary informed consent forms signed by the research participants have been obtained. The relevant content and purpose of the study were within the scope of standardized informed consent. The questionnaire survey is mainly aimed at adults. Although children, as part of the souvenir market, have interests and preferences that have an important impact on certain types of products (e.g., cartoon image souvenirs), children do not the ability to consume directly. Therefore, this study focuses more on exploring and understanding adult consumer preferences and behaviors, in order to provide companies with more accurate market insights and strategic recommendations, and promote the healthy development of the souvenir market.

To ensure the objectivity and reliability of the satisfaction and aesthetic evaluation results for these 10 tourism mascots, the analysis employed a One-Way ANOVA test to assess the significance of differences in the evaluation outcomes for each mascot. The F-value of the test statistic was calculated, and the P-value was determined to facilitate statistical inference. This approach ensures that the differences in the evaluation results across the various tourism mascots are rigorously tested for significance, thereby supporting the credibility of the findings.


$$F=U/r1/\left(V/r2\right)$$ (1)

where U and V represent the degrees of freedom corresponding to *r*1 and *r*2, respectively. if the degrees of freedom for the F-statistic lie between *r*1 and *r*2, then the F-statistic will follow the distribution described by the following equation:


$$h\left(w\right)=\frac{\Gamma \left(\frac{r1+r2}{2}\right){\left(\frac{r1}{r2}\right)}^{\frac{r1}{2}}W^{\frac{r1}{2}-1}}{\Gamma \left(\frac{r1}{2}\right)\Gamma \left(\frac{r2}{2}\right){\left(1+\frac{r1\text{w}}{r2}\right)}^{\frac{r1+r2}{2}}}$$ (2)

This equation describes the probability density function of the F-distribution, which is used to determine the likelihood of observing a given F-value under the null hypothesis.

By evaluating the reliability of the variables within the tourism mascot assessment questionnaire, we can ascertain the validity and soundness of the questionnaire design. In this study, IBM SPSS Statistics for MAC was utilized to conduct the statistical analyses, including frequency analysis, descriptive statistical analysis, and one-way ANOVA. The threshold for statistical significance (p-value) was set at 0.05. Detailed statistical results are presented in Table 2.

**Table 2:** Statistical analysis method.

Distinguish		Content	Remark
Analyze	General Questionnaire	Frequency and mean analysis	IBM SPSS Statistics for MAC
	Questionnaire reliability analysis	Quantitative data Cronbach.α coefficient value analysis	
	Cultural and creative brand mascot analysis (Satisfaction analysis, aesthetic semantic analysis)	Frequency analysis, analysis of variance (ANOVA - one-way analysis of variance), is used to analyze the relationship between categorical and quantitative data.	

### 2.5. Respondent demographics

A total of 226 questionnaires were distributed in this survey, with 205 valid responses collected, resulting in a response rate of 90.7%. The demographic analysis of the respondents is as follows: 41.95% were male, and 58.05% were female. Age distribution showed that 14.63% of respondents were between 18–20 years old, 39.51% were 21–30 years old, 32.68% were 31–40 years old, 9.76% were 41–50 years old, and 3.41% were over 51 years old.

Regarding educational attainment, 7.32% of respondents had an education level below high school, 8.78% were junior college students, 57.56% were undergraduates, 10.73% were master’s students, and 15.61% were doctoral students. Occupationally, 44.88% of respondents were full-time students, 21.95% were employed in institutions or as civil servants, 18.05% were company employees, 3.9% were self-employed, 5.37% were freelancers, and 5.85% belonged to other occupations. In terms of income level, 30.73% of respondents had an income below 1,000, 34.15% earned between 1,000–5,000, 20% had an income of 5,000–10,000, 9.76% earned between 10,000–15,000, and 5.37% had an income above 15,000.

These demographic insights provide a comprehensive understanding of the survey participants and ensure the representativeness of the data collected.

### 2.6. Reliability analysis

The reliability of the Xi’an tourism mascots evaluation questionnaire was assessed using SPSS software. The reliability of the questionnaire was determined by analyzing the Cronbach’s α coefficient, which reflects the internal consistency of the scale. A Cronbach’s α value in the range of 0.8 to 0.9 indicates a high-level of reliability. The questionnaire contains13 items for evaluating the tourism mascots. The analysis yielded the following Cronbach’s α values for each mascot: X1 (0.945), X2 (0.968), X3 (0.980), X4 (0.979), X5 (0.984), X6 (0.986), X7 (0.985), X8 (0.980), X9 (0.971), and X10 (0.982). These results demonstrate that the Cronbach’s α values for all tourism mascots exceed 0.9, indicating that the questionnaire is highly reliable and effective in evaluating the intended constructs.

## 3. Survey results

### 3.1. Satisfaction evaluation

Question: Are you satisfied with the design of these tourism mascots, as shown in Table 3.

**Table 3:** From 1 to 7 indicate extremely dissatisfied at extremely satisfied.

Dissatisfied	1	2	3	5	6	7	Satisfied

As shown in Table 4, an analysis of the satisfaction survey results for the 10 Xi’an tourism mascots reveals that the average satisfaction ratings for all mascots fall within the ‘4–6’ interval. This range indicates that overall satisfaction levels are between ‘average’ and ‘quite satisfied,’ suggesting a generally positive evaluation of the mascot designs.

**Table 4:** Satisfaction evaluation results.

		N	Average value	Standard deviation	F	P
Satisfaction	X1	205	4.99	1.368	22.289	0.000
	X2	205	4.26*	1.589		
	X3	205	5.09	1.434		
	X4	205	5.62	1.233		
	X5	205	4.71	1.496		
	X6	205	4.74	1.484		
	X7	205	4.62	1.701		
	X8	205	5.31	1.465		
	X9	205	5.85**	1.176		
	X10	205	4.86	1.541		

*Indicates the lowest score for satisfaction.

**Indicates the highest score for satisfaction.

The order of satisfaction, from highest to lowest, is as follows: X9 >  X4 >  X8 >  X3 >  X1 >  X10 >  X6 >  X5 >  X7 >  X2. X9 received the highest satisfaction score, while X2 had the lowest. The results of the one-way ANOVA further support these findings, with an F-value of 22.289 and a P-value of 0.000, which is less than the significance threshold of 0.05. This indicates a statistically significant difference in satisfaction levels among the ten groups, suggesting that tourism mascots, which are based on different regional cultures, significantly impact consumer satisfaction.

### 3.2. Aesthetic sensitivity evaluation

Question: Use the following adjectives to rate as shown in the picture, as shown in Table 5.

**Table 5:** From rang of −3 to 3 indicates extremely negative to extremely positive.

Tradition	−3	−2	−1	0	1	2	3	Modern

As presented in Table 6, the SD (Semantic Differential) evaluation results for each mascot were analyzed using one-way ANOVA to calculate the mean and standard deviation. The analysis revealed that the standard deviations were generally above 1, indicating a certain degree of dispersion in the data. This suggests that there were noticeable differences in the measurement scores for each mascot, reflecting low similarity among the various tourism mascots. These results underscore the diversity in consumer perceptions and evaluations, highlighting the distinctiveness of each mascot design.

**Table 6:** One way ANOVA of aesthetic sensitivity evaluation.

Content			X1	X2	X3	X4	X5	X6	X7	X8	X9	X10	F	P
Market positioning	Traditional – Modern	Average	0.35	0.50	0.84*	1.32	0.55*	0.47*	0.73	0.88	1.84	1.00	16.150	.000
		Std. Deviation	1.767	1.691	1.519	1.486	1.655	1.641	1.724	1.742	1.231	1.643		
	Ordinary – Innovative	Average	0.60	0.53	0.93	1.47	0.57	0.52	0.69	1.15	1.88**	0.93	17.031	.000
		Std. Deviation	1.703	1.696	1.503	1.385	1.625	1.592	1.782	1.557	1.239	1.715		
Market trends	Outdated – Popular	Average	0.96	0.34	1.00	1.38	0.66	0.64	0.66	1.18	1.80	0.95	15.388	.000
		Std. Deviation	1.546	1.587	1.587	1.386	1.581	1.586	1.718	1.540	1.262	1.650		
	Rational – Emotional	Average	0.53	0.52	1.07	1.42	0.73	0.60	0.68	1.15	1.66	0.85	14.153	.000
		Std. Deviation	1.516	1.481	1.356	1.321	1.513	1.514	1.695	1.534	1.332	1.632		
Color decoration	Plain – Gorgeous	Average	0.03*	0.50	1.05	1.41	0.80	0.61	0.68	1.35**	1.70	0.98	21.023	.000
		Std. Deviation	1.652	1.697	1.471	1.389	1.517	1.570	1.676	1.480	1.251	1.634		
	Cool – Warm	Average	0.92	0.39	1.06	1.45	0.70	0.65	0.61	1.34	1.77	0.98	16.906	.000
		Std. Deviation	1.382	1.628	1.423	1.315	1.548	1.509	1.681	1.508	1.238	1.622		
	Hard – Soft	Average	0.99	0.47	1.15**	1.51	0.70	0.65	0.48*	1.18	1.83	0.91	18.111	.000
		Std. Deviation	1.352	1.673	1.393	1.282	1.567	1.550	1.647	1.527	1.189	1.638		
Form features	Uninteresting – Interesting	Average	0.90	0.49	1.07	1.57	0.69	0.70	0.60	1.22	1.87	1.07**	17.858	.000
		Std. Deviation	1.435	1.697	1.460	1.273	1.546	1.517	1.670	1.494	1.187	1.570		
	Complex – Simple	Average	0.83	0.91**	1.04	0.96*	0.70	0.72	0.65	0.51*	0.71*	0.55*	2.690	.004
		Std. Deviation	1.261	1.394	1.244	1.445	1.481	1.430	1.622	1.762	1.752	1.725		
	Vulgar – Noble	Average	0.58	0.23*	0.95	1.25	0.66	0.53	0.52	1.20	1.55	0.79	16.336	.000
		Std. Deviation	1.268	1.525	1.376	1.289	1.521	1.487	1.583	1.463	1.214	1.603		
Psychological feelings	Negative – Positive	Average	1.25**	0.71	1.11	1.59**	0.81**	0.76**	0.75**	1.30	1.73	1.01	12.674	.000
		Std. Deviation	1.299	1.591	1.364	1.256	1.506	1.462	1.642	1.454	1.292	1.605		
	Meaningless – Meaningful	Average	1.08	0.45	1.05	1.47	0.66	0.68	0.71	1.26	1.69	0.92	14.451	.000
		Std. Deviation	1.378	1.655	1.441	1.285	1.540	1.538	1.597	1.478	1.252	1.595		

*Indicates the lowest score of tourism mascots.

**Represents the highest score of tourism mascots.

Based on the statistical results, the five tourism mascots—X4, X1, X5, X6, and X7—scored highest in the “Negative - Positive” measurement. These mascots draw inspiration from historical figures and cultural relics, such as the Tang Dynasty figurines, Emperor Xuanzong of Tang, the Yulin Army, and the Terracotta Warriors from the Qin culture. The high scores in this dimension suggest that these mascots, deeply rooted in historical and cultural heritage, evoke a strong sense of cultural pride and self-confidence among consumers. Mascot X2 achieved the highest score in the “Complex - Simple” dimension. Its design is intricately linked to cultural allusions from *The Analects of Confucius*, specifically Yanhui’s teachings of “See no evil, hear no evil, speak no evil, do not act evil.” The nuanced expressions of the characters effectively convey the gentle and refined essence of Confucian culture centered around ritual and music. However, the predominance of monochromatic colors in X2’s design leaves a relatively simple impression in terms of color decoration. X3 excelled in the “Hard - Soft” dimension, with its overall image characterized by a simple red outfit and flushed cheeks, presenting a lively and endearing appearance. This combination likely contributes to a soft and approachable perception among consumers. Mascot X8 garnered the highest score in the “Plain - Gorgeous” measurement, attributed to the rich incorporation of dramatic elements in its design. The lifelike character depictions and vibrant colors of X8 stand out as more ornate compared to other mascots, contributing to its perceived grandeur. X9 stood out in the “Ordinary - Innovative” dimension, largely due to its fusion of historical and cultural elements with animal imagery, employing personification in a novel manner. The mascot’s expressive body language, vivid facial expressions, smooth lines, and harmonious color scheme create a compelling and innovative design. Additionally, the culturally meaningful attire and accessories imbue the mascot with a narrative richness. Finally, X10 received the highest score in the “Uninteresting - Interesting” dimension. The mascot’s design, characterized by a simple, rounded, and energetic form, contrasts sharply with the more familiar agile form of horses. The unique approach to color, pattern decoration, and material texture distinguishes X10 from conventional realistic animal images, offering a fresh and intriguing appeal.

In contrast, X3, X5, and X6 scored the lowest in the “Traditional - Modern” measurement. This outcome may be attributed to their limited variability in form and the lack of a compelling narrative element. However, this also suggests a high degree of alignment with the traditional cultural image of Xi’an in the minds of consumers. While these mascots may not embody modernity in terms of market positioning, they play a vital role in promoting Xi’an’s regional culture and representing its cultural identity. Conversely, X4, X8, X9, and X10 received the lowest scores in the “Complex - Simple” dimension. X1 ranked lowest in the “Plain - Gorgeous” dimension, X2 in the “Vulgar - Noble” dimension, and X7 in the “Hard - Soft” dimension. These results are primarily due to the design elements incorporated into X1 and X2, which draw from the Terra Cotta Warriors of the Qin Dynasty, and X7, which is inspired by the visual elements of the Tang Dynasty’s imperial military road. These mascots are all modeled after battle-hardened and majestic soldiers. Designing them with noble, gorgeous, or soft visual elements would contradict their historical and cultural significance. Instead, using visual expressions that emphasize honesty, simplicity, strength, and resolve highlights the intrinsic characteristics of these mascots, maintaining their authenticity and reinforcing their cultural resonance.

**Fig 3:**
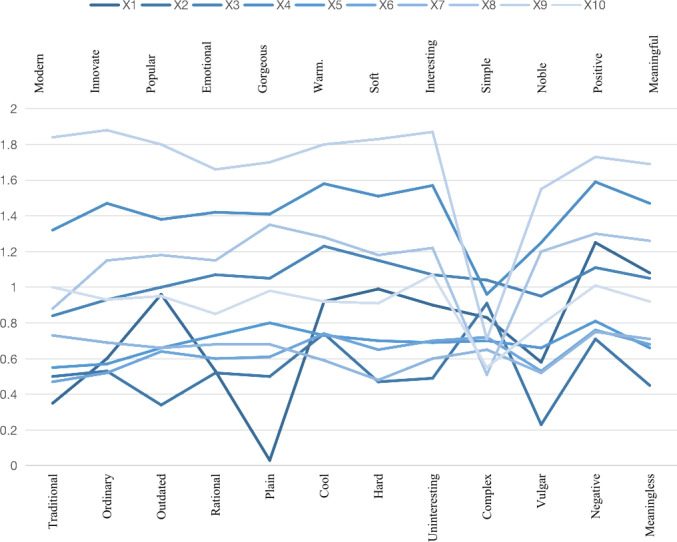
Semantic differential (SD) evaluation curve of tourism mascots.

As illustrated in Fig 3, the scores for all 10 tourism mascots are positive, indicating a generally favorable evaluation and a certain degree of reliability in the assessment. Moreover, these scores exhibit noticeable differences among the mascots, suggesting variability in consumer perceptions. Overall, the evaluation results derived from the Semantic Differential (SD) method are statistically significant, reinforcing the robustness of the findings.

## 4. Discussion

**Fig 4:**
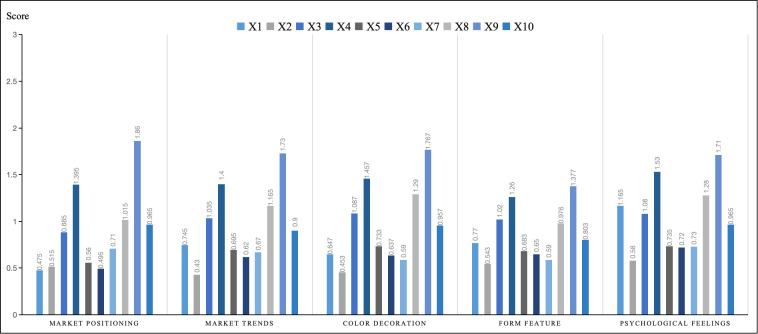
Average evaluation of tourism mascots using the semantic differential (SD) method across five dimensions.

The aesthetic evaluation model for tourism mascots developed in this study incorporates five dimensions: market positioning, market trends, color decoration, styling features, and psychological feelings. The average Semantic Differential (SD) scores for each of the 10 tourism mascot images were calculated across these dimensions, and the results are illustrated in the score chart presented in Fig 4. Statistical analysis reveals that the top three mascots in terms of comprehensive evaluation across these dimensions are X9, X4, and X8. These rankings align with the top three satisfaction scores, suggesting that the SD evaluation results are both rational and credible. This consistency underscores the reliability of the aesthetic evaluation model and its effectiveness in capturing consumer perceptions of the mascots.

From the perspective of market positioning, the top two mascots, X9 and X4, which incorporate elements of Tang culture in their design, stand out in the statistical results. Tang culture, a significant representative of Xi’an’s regional heritage, was characterized by its advanced development in economy, culture, technology, and art, fostering cross-ethnic exchanges and assimilating foreign influences. This rich and diverse cultural background plays a crucial role in bridging historical and contemporary aesthetics. The inclusiveness and openness of Tang culture resonate with modern social values, including popular elements such as Tang suits and accessories, making it particularly appealing and recognizable to young consumers. In contrast, the Qin culture, represented by the Terracotta Warriors, should delve deeper into its core cultural elements and align with future market trends to strengthen the development of mascots. By integrating traditional visual elements with contemporary design trends, mascots inspired by Qin culture could achieve greater relevance and appeal.

From the perspective of market trends, the innovative transformation of regional cultural elements must take into account the aesthetic preferences of younger consumer groups. Tourism mascots are designed to fulfill consumers’ spiritual and cultural needs. Mascots that align with these emotional and cultural needs are more likely to resonate with consumers compared to those designed primarily from the viewpoint of regional cultural promotion. The survey results of the market trend dimension, specifically the adjectives ‘Outdated - Popular’ and ‘Rational - Emotional,’ show a trend toward favoring mascots that evoke strong emotional connections and rich storytelling. This indicates that mascots with an emotional appeal and fascinating storylines are more popular with consumers. Consequently, when designing tourism mascots, it is crucial to focus on market orientation and integrate it with regional cultural promotion. Relying solely on the concept of promoting regional culture may not be sufficient to create mascots that evokes a strong market response.

From the perspective of color decoration, the design of tourism mascots must consider not only the product features but also cultural background, market trends, and production techniques. The integration of diverse colors is often more visually appealing than a single-color scheme, resulting in a fuller and more vivid image. For example, the X4 mascot, inspired by the “maid figurines” collection at the Xi’an History Museum, exemplifies effective use of color and decoration. The design team meticulously studied the makeup, hair accessories, and clothing of Tang Dynasty girls, combining these historical elements with modern design techniques and engaging design language. This approach successfully restored and highlighted the confident and charming image of Tang Dynasty girls, applying it creatively to various mascot designs.

From the perspective of modeling, which involves shaping the unique image of an object, this element is crucial in the design of cultural and creative products. Modeling is not only essential for creating the most immediate and intuitive impression on consumers but also plays a significant role in integrating regional cultural elements into these designs. Through visual and tactile experiences, well-crafted modeling can subtly convey cultural connotations, enhancing the overall creative impact of the product. Tourism mascots with engaging and distinctive designs do more than capture attention; they leave a lasting impression. The interplay between appearance and demeanor is vital, as capturing the intended demeanor and combining it with aesthetically pleasing forms can better showcase product features and communicate cultural meanings. For instance, the modeling of X9 effectively conveys the essence of Tang culture in Xi’an. By employing metaphors and incorporating decorations and colors that align with the characteristics of Tang Dynasty aesthetics, the design achieves a harmonious balance between surface decoration and modeling, resulting in a more coherent and appealing product image. Adding lively and interesting elements to the modeling by reinterpreting culture, cultural relics, and traditional craftsmanship, combined with modern creativity, can meet consumers’ visual expectations while imbuing the product with deeper cultural significance. For example, X4 and X8 adopt a Q-version style with exaggerated proportions ranging from 1:1 to 2:1, which enhances their lively and charming appearance. The dynamic, life-like features of these mascots create a vivid, engaging presence that feels both comfortable and familiar. Therefore, an excellent mascot design harmonizes form and spirit, capturing the essence of both to create a compelling and meaningful product.

From the perspective of psychological perception, the design of an effective tourism mascot should transcend mere representations of historical figures or natural elements. It should foster an interaction that nurtures emotional connections, allowing the mascot to evoke cherished memories, enhance the aesthetic experience, and create a sense of familiarity and joy. For instance, the X9 mascot design taps into consumers’ affection for pets by anthropomorphizing dynamic and lifelike cat images, aligning with consumer psychology. Similarly, X4 is designed with a relaxed and casual demeanor, resonating with young consumers and fostering empathy through its portrayal of a zest for life. This illustrates that by addressing emotional needs and strategically applying psychological principles, the design of tourism mascots can significantly contribute to brand development and is more likely to deliver unexpected enjoyment and engagement for consumers.

## 5. Conclusion

This study evaluates the satisfaction and aesthetic evaluation of cultural and creative products in Xi’an based on the semantic difference method, and combines frequency analysis, one-way ANOVA methods, and scoring standard analysis to draw the following conclusions:

1. The aesthetic semantic evaluation model for tourism mascots based on regional culture is composed of five dimensions: market positioning, market trends, color decoration, form features, and psychological perception. These dimensions reflect the evaluation of cultural and creative brand mascots in terms of innovation, trends, appearance, market relevance, and consumer perception. The model is further divided into 12 evaluation factors, establishing a comprehensive framework for assessing tourism mascots.
2. By analyzing consumer characteristics, this study obtained evaluation results regarding consumer satisfaction with tourism mascots based on Xi’an regional culture, verifying the correlation between consumers’ aesthetic assessments and satisfaction with mascot images across five dimensions. The questionnaire survey also revealed a 1.5-fold increase in the number of consumers willing to purchase cultural and creative products after learning about Xi’an tourism mascots. This finding indicates that most consumers have a positive evaluation of the current design of Xi’an tourism mascots and are inclined to continue exploring and purchasing these products.
3. After an in-depth analysis of the analysis results, it is clear that color and shape constitute key dimensions in assessing the aesthetic value of a mascot image’s appearance. It is true that the material, texture and material of the product also have a significant impact on the consumer’s purchase intention, but from the perspective of aesthetic evaluation, color and shape are the first and main elements to attract visual attention. In comparison, material texture and texture characteristics are more related to the level of tactile experience. They are usually examined after the initial visual perception as a complementary means to further deepen the understanding of product material. This process presents a progressive relationship from vision to touch. Therefore, in the evaluation of tourism mascot satisfaction, those works with harmonious color combinations, unique shape design and “both spirit and form” – that is, the perfect integration of external form and internal symbolism – will often receive higher scores. This finding highlights the central role of visual aesthetics in initially capturing consumers’ attention and forming a positive impression, while also pointing to the importance of touch in deepening product perception in subsequent experiences, but all of this is based on the appeal of visual aesthetics.
4. To a certain extent, the evaluation of tourism mascots is positively correlated with the degree of expression of the regional cultural connotations contained in them. When the aesthetic concepts of Tang culture are integrated into the mascot design, the recognizability and cultural appeal of the tourism mascot image can be significantly improved. Tang culture is famous for its rich visual expression. If the essence and characteristics of Tang culture can be fully displayed in the image design of mascots, it will not only give the tourism mascots a deeper cultural connotation, but also more effectively spread the Xi’an regional culture. In contrast, the manifestation of Qin culture relies more on the widespread influence of the Terracotta Warriors and Horses, which in turn drives the creation and derivation of related tourist souvenirs. However, in terms of the visual expression of cultural products, Qin culture appears to be relatively homogenous. As for other cultural elements, although they may be interesting to a certain extent, they are often not as vivid and prominent as the Tang and Qin cultures in expressing the depth of Xi’an’s regional culture. Through in-depth exploration and clever use of the essence of regional culture, especially the rich visual expression and profound cultural connotation of cultural resources such as Tang culture, the cultural value and aesthetic appealing of tourism mascots can be significantly enhanced, thereby better disseminating and promoting regional culture.

The evaluation of the aesthetic sensitivity of tourism mascots based on the semantic differential method also has certain limitations. For example, the selection of adjective pairs may be subjective and one-sided, thus affecting the accuracy and objectivity of the evaluation results. Factors such as the cultural background, education level, and aesthetic experience of the respondents may also affect their aesthetic evaluation of tourism mascots. At the same time, targeted surveys and analyses are needed for different audience groups to better meet market and audience needs.

In summary, for tourism mascots based on regional culture to excel as cultural and creative brands, it is essential to first understand market demand and trends, and integrate regional cultural elements with public aesthetics. This approach ensures that regional cultural characteristics are maximized, resulting in mascots with rich designs, distinctive features, and high recognizability. Offering consumers valuable and meaningful cultural and creative products is crucial for advancing the development of cultural and creative brands. This study found that the use of semantic differential method to evaluate the aesthetic sensitivity of tourism mascots has obvious advantages such as comprehensiveness, innovation, and strong scientificity, but it also has its limitations, mainly reflected in the subjectivity of the research itself, and the influence of cultural, educational, aesthetic and other factors of different audience groups, which may affect the research results. Therefore, in practical applications, it is necessary to flexibly select and adjust the evaluation method to ensure the effectiveness and practicality of the evaluation. Future research will continue to explore more effective evaluation methods and optimization strategies, contributing further to the advancement of the cultural tourism industry.

## References

[1] Tajeddini K, Martin E, Altinay L. The importance of human-related factors on service innovation and performance. Int J Hosp Manag. 2020;85:102431. doi: 10.1016/j.ijhm.2019.102431

[2] Chiou SC, Wang YC. The example application of genetic algorithm for the framework of cultural and creative brand design in Tamsui Historical Museum. Soft Comput. 2018;22(8):2527–45. doi: 10.1007/s00500-017-2507-9

[3] Zhu W, Han S. [Retracted] Cultural product appearance design based on improved multiobjective optimization algorithm. Secur Commun Networks. 2022;2022(1):1–9. doi: 10.1155/2022/7063303

[4] Li M, Liu Y. [Retracted] The impact of cultural creative product design for sport events on the residents’ fitness. J Environ Public Health. 2022;2022(1):3150099. doi: 10.1155/2022/3150099 36120142 PMC9477587

[5] Zhang W. Study on the current situation and countermeasures of the history of health culture development in Shaanxi based on the background of big data. Appl Math Nonlinear Sc. 2024;9(1): doi: 10.2478/amns.2023.2.00318

[6] Dai Y. Application of regional culture in landscape architecture design under the background of data fusion. Sci Program. 2022;2022(1):1–12. doi: 10.1155/2022/6240313

[7] Zhang A, Yang Y, Chen T, Liu J, Hu Y. Exploration of spatial differentiation patterns and related influencing factors for National Key Villages for rural tourism in China in the context of a rural revitalization strategy, using GIS-based overlay analysis. Arabian J Geosci. 2021;14(2):83. doi: 10.1007/s12517-020-06381-9

[8] Aalders I, Stanik N. Spatial units and scales for cultural ecosystem services: a comparison illustrated by cultural heritage and entertainment services in Scotland. Landsc Ecol. 2019;34(7):1635–51. doi: 10.1007/s10980-019-00827-6

[9] Yang W, Lin B, Wen C. Cultural characteristics and geospatial distribution of landscape ecology in the perspective of regional culture. Arabian J Geosci. 2021;14(22):2264. doi: 10.1007/s12517-021-08623-w

[10] Roy Chowdhury R, Turner Ii BL. The parallel trajectories and increasing integration of landscape ecology and land system science. J Land Use Sci. 2019;14(2):135–54. doi: 10.1080/1747423X.2019.1597934

[11] Wang Q, H A. Design of watercolor cultural and creative products based on style transfer algorithm. Math Probl Eng. 2022;2022(1):1–9. doi: 10.1155/2022/2711861

[12] Qiu Song QQ. Exploration of design methodology of the brand-driven cultural and creative products. Packag Eng. 2019;24:11–7.

[13] Qingqing Z. Research on the innovation path of Lingbi stone culture based on cultural semiotics. Art Perform Lett. 2023;4(10):39–44.

[14] Sun Ai-hui CD. The application value of IP image in brand communication. Designs. 2019;10:66–7.

[15] Wang J, Cheng F, Chen C. Optimization and evaluation of tourism mascot design based on analytic hierarchy process–entropy weight method. Entropy. 2024;26(7):585. doi: 10.3390/e26070585 39056947 PMC11276575

[16] Wang Y, Zhao Q, Chen J, Wang W, Yu S, Li C, et al. Perceptual quantitative decision making and evaluation of product stylable topology design. Processes. 2022;10(9):1819. doi: 10.3390/pr10091819

[17] Sumi M. Simulation of artificial intelligence robots in dance action recognition and interaction process based on machine vision. Entertainment Comput. 2025;52:100773. doi: 10.1016/j.entcom.2024.100773

[18] Zhong Ming ZZ, Zhang C-hui, Xiaoyong Li. The aesthetic semantic model of mountain scenic spot landscape: a case study of Taibai mountain. J Zhejiang Univ (Sci Ed). 2021;48(3):368–76.

[19] Bradley MM, Lang PJ. Measuring emotion: the self-assessment manikin and the semantic differential. J Behav Ther Exp Psychiatry. 1994;25(1):49–59. doi: 10.1016/0005-7916(94)90063-9 7962581

[20] Takahashi H, Ban M, Asada M. Semantic differential scale method can reveal multi-dimensional aspects of mind perception. Front Psychol. 2016;7:1717. doi: 10.3389/fpsyg.2016.01717 27853445 PMC5090820

[21] Takahashi H, Terada K, Morita T, Suzuki S, Haji T, Kozima H, et al. Different impressions of other agents obtained through social interaction uniquely modulate dorsal and ventral pathway activities in the social human brain. Cortex. 2014;58:289–300. doi: 10.1016/j.cortex.2014.03.011 24880954

[22] Su Chen ZX, Jiayong Z. Research on imitation furniture design based on extension semantics and shape grammar. For Ind. 2023;60(11):58–65.

[23] Fu Hao LR, Boxun W. Research on the influencing factors of attachment emotions in the design of rural public buildings in Lingnan. Furniture Inter Decoration. 2022;12:112–7.

[24] Child IL. Observations on the meaning of some measures of esthetic sensitivity. J Psychol. 1964;57(1):49–64. doi: 10.1080/00223980.1964.9916671 14100130

[25] Eysenck HJ. The general factor in aesthetic judgements. Br J Psychol Gen Sec. 1940;31(1):94–102. doi: 10.1111/j.2044-8295.1940.tb00977.x

[26] Eysenck HJ. A new measure of ‘Good Taste’ in visual art. Leonardo. 1983;16(3):229–31. doi: 10.2307/1574921

[27] Myszkowski N. The first glance is the weakest: “Tasteful” individuals are slower to judge visual art. Pers Indiv Diff. 2019;141:188–95. doi: 10.1016/j.paid.2019.01.010

[28] Myszkowski N, Çelik P, Storme M. A meta-analysis of the relationship between intelligence and visual “taste” measures. Psychol Aesthet Creat the Arts. 2018;12(1):24–33. doi: 10.1037/aca0000099

[29] Marschallek BE, Weiler SM, Jörg M, Jacobsen T. Make it special! Negative correlations between the need for uniqueness and visual aesthetic sensitivity. Empir Stud Arts. 2021;39(1):101–17. doi: 10.1177/0276237419880298

[30] Duffy RA. An analysis of aesthetic sensitivity and creativity with other variables in grades four, six, eight, and ten. J Educ Res. 1979;73(1):26–30. doi: 10.1080/00220671.1979.10885199

[31] Bairisal SKA. Response time differences in the aesthetic judgment of individuals on beautiful and ugly images. Int J Arts Humanit Soc Sci Stud. 2020;5:19–29.

[32] Keltner D, Haidt J. Approaching awe, a moral, spiritual, and aesthetic emotion. Cogn Emot. 2003;17(2):297–314. doi: 10.1080/02699930302297 29715721

[33] Thrash TM, Elliot AJ. Inspiration as a psychological construct. J Pers Soc Psychol. 2003;84(4):871–89. doi: 10.1037/0022-3514.84.4.871 12703654

[34] Atari M, Afhami R, Mohammadi-Zarghan S. Exploring aesthetic fluency: the roles of personality, nature relatedness, and art activities. Psychol Aesthet Creat Arts. 2020;14(1):125–31. doi: 10.1037/aca0000200

[35] Silvia PJ, Fayn K, Nusbaum EC, Beaty RE. Openness to experience and awe in response to nature and music: personality and profound aesthetic experiences. Psychol Aesthet Creat Arts. 2015;9(4):376–84. doi: 10.1037/aca0000028

[36] Afhami R, Mohammadi-Zarghan S. The Big Five, Aesthetic Judgment Styles, and Art Interest. Europe’s J Psychol. 2018;14(4):764–75. doi: 10.5964/ejop.v14i4.1479 30555584 PMC6266521

[37] Myszkowski N. “Aesthetic sensitivity.” In: The Oxford Handbook of Empirical Aesthetics. Oxford University Press; 2022.

[38] Wanzer DL, Finley KP, Zarian S, Cortez N. Experiencing flow while viewing art: development of the aesthetic experience questionnaire. Psychol Aesthet Creat Arts. 2020;14(1):113–24. doi: 10.1037/aca0000203

[39] Jacobi CJ, Varga PJ, Vaidyanathan B. Aesthetic experiences and flourishing in science: a four-country study. Front Psychol. 2022;13:923940. doi: 10.3389/fpsyg.2022.923940 36017445 PMC9396270

[40] Zhang J, Lu Z, Wang Y, Bai X. The aesthetic developmental characteristics of contour features in children and adolescents with high- and low- level visual aesthetic sensitivity across grade levels. Behav Sci. 2024;14(5):416. doi: 10.3390/bs14050416 38785908 PMC11117876

[41] Ghadiri SM, Zhuge Y. Urban construction and management engineering IV. CRC Press; 2024.

[42] Yao Xiang HH. Cultural and creative product design. Peking University Press; 2021.

[43] Lei S. The value and design of cultural and tourism mascots. Art Observ. 2019;12:23–4.

[44] Zhixin L. Research on the design of tourism mascot in Ji’an City - combining the successful experience of Kumamoto in Japan. Cult J. 2021;2:122–4.

[45] Wu Y. Evolutionary design method for transforming ceramic cultural elements into cultural and creative products. J Intell Fuzzy Syst. 2023;45(5):7297–315. doi: 10.3233/jifs-231906

