# Expression of Concern: Research on the aesthetic sensitivity evaluation of tourism mascots based on semantic differential method

**DOI:** 10.1371/journal.pone.0343103

**Published:** 2026-02-17

**Authors:** The PLOS One Editors

After this article [1] was published, the following concerns were noted:

- The articles cited as References 4, 5, 10, 17, and 21 appear to be irrelevant to this article [1].
- The articles cited as References 3, 4, and 45 were retracted before [1] was published

We regret that the issues were not identified prior to the article’s publication.

In light of these concerns, the *PLOS One* Editors issue this Expression of Concern. Readers are advised to interpret the article [1] with caution.
